# A novel method for precise implantation of tracheal Y-shaped stent

**DOI:** 10.3389/fmed.2024.1337669

**Published:** 2024-04-08

**Authors:** Xin-Min Ding, Yu-An Ding, Yan-Fang Duan, Jiao-Yang Chen, Long Li, Fang-Ping Ren, Jie Sun

**Affiliations:** ^1^Department of Respiratory and Critical Care Medicine, Beijing Shijitan Hospital Affiliated to Capital Medical University, Beijing, China; ^2^Beijing Bayi School, Beijing, China

**Keywords:** tracheal Y-shaped stent, precise implantation, double-lumen endotracheal intubation, airway stenosis, esophagotracheal fistula

## Abstract

The tracheal Y-shaped stent is mainly used for the treatment of critical patients with airway stenosis or esophagotracheal fistula near carina. A novel method for precise implantation of Y-shaped tracheal stents was developed using double-lumen endotracheal intubation and flexible bronchoscopy. This approach aims to address the limitations associated with X-ray or rigid bronchoscopy guidance, such as operational difficulties and the risk of inaccurate stent placement leading to implantation failure or suffocation. With this new technique, 13 tracheal Y-shaped stents were successfully implanted. This method shows promise in reducing the complexity of stent implantation and facilitating timely treatment for patients in need. Additionally, it has the potential to update current operating standards and guidelines for this procedure.

## Introduction

The tracheal Y-shaped stent is mainly used for the treatment of critical patients with airway stenosis or esophagotracheal fistula near carina (1, 2). Currently, the implantation of tracheal Y-shaped stent is predominantly guided by X-ray (3–5) or rigid bronchoscopy (6–10) poses operational difficulties and can result in imprecise positioning, leading to implantation failure or even asphyxia. To overcome the above shortcomings, a double-lumen endotracheal intubation had developed for precise implantation of tracheal Y-shaped stent, which has been granted a Chinese patent (patent number: 202321038543.3). This innovation allows the implantation of the tracheal Y-shaped stent to be performed under direct visualization with a bronchoscope, reducing the difficulty of operation and improving the implantation accuracy. We have successfully implanted 13 tracheal Y-shaped stents using this method. The following are two representative cases.

## Case reports

### Case 1

**Figure 1:**
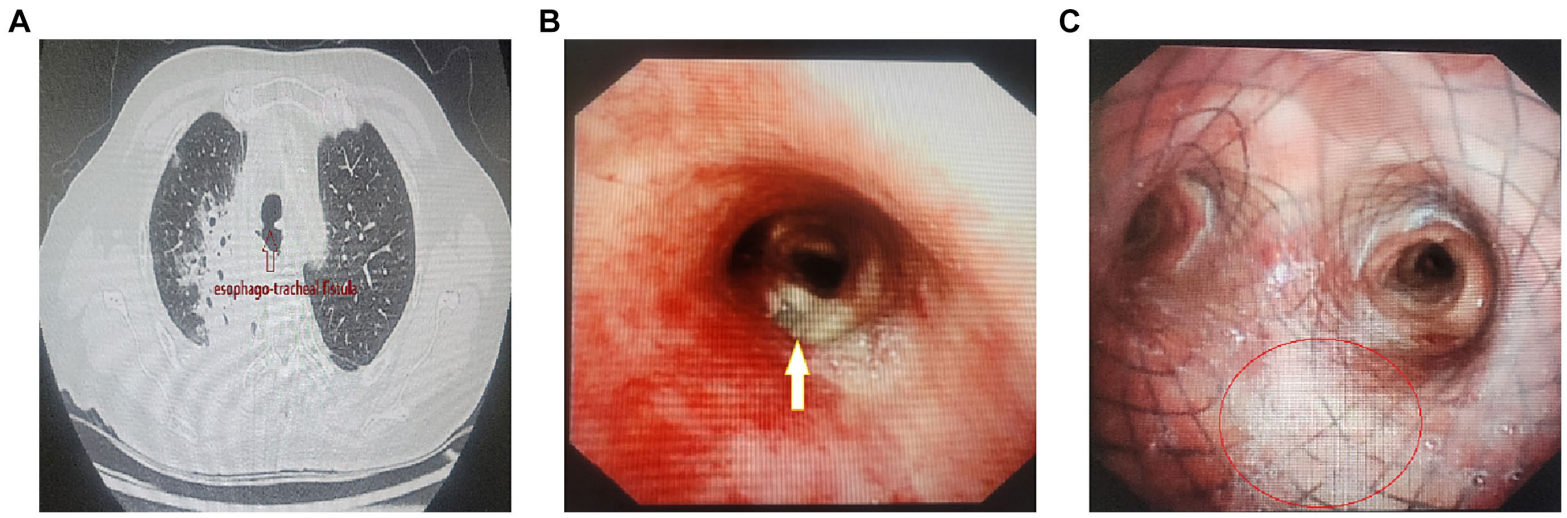
**(A)** CT shows esophago-tracheal fistula (↑) with aspiration pneumonia of the right lung. **(B)** Bronchoscopy reveals a 2 cm diameter esophageal-tracheal fistula adjacent to the right main bronchus in close proximity to the carina (↑). **(C)** The fistula (○) was sealed by self-expanding Y-shaped metal covered stent.

A 61-year-old woman (Identification number 501706) was admitted to our hospital due to an esophagealtracheal fistula following postoperative radiotherapy for lung cancer, along with a secondary pulmonary infection and dyspnea. Upon admission, chest CT scan and bronchoscopy revealed a 2 cm diameter esophagealtracheal fistula near the carina. The fistula allowed gastric contents to pass into the lungs (Figures 1A,B). As a treatment, a customized self-expanding Y-shaped metal covered stent (Micro-Tech Medical Company, Nanjing, China) was precisely implanted in the patient’s airway to effectively seal the fistula (Figure 1C). In order to reduce the stimulation of the fistula by food and provide nutritional support to the patient, a jejunostomy was also performed. The details of the stent implantation procedure are described below.

### Case 2

**Figure 2:**
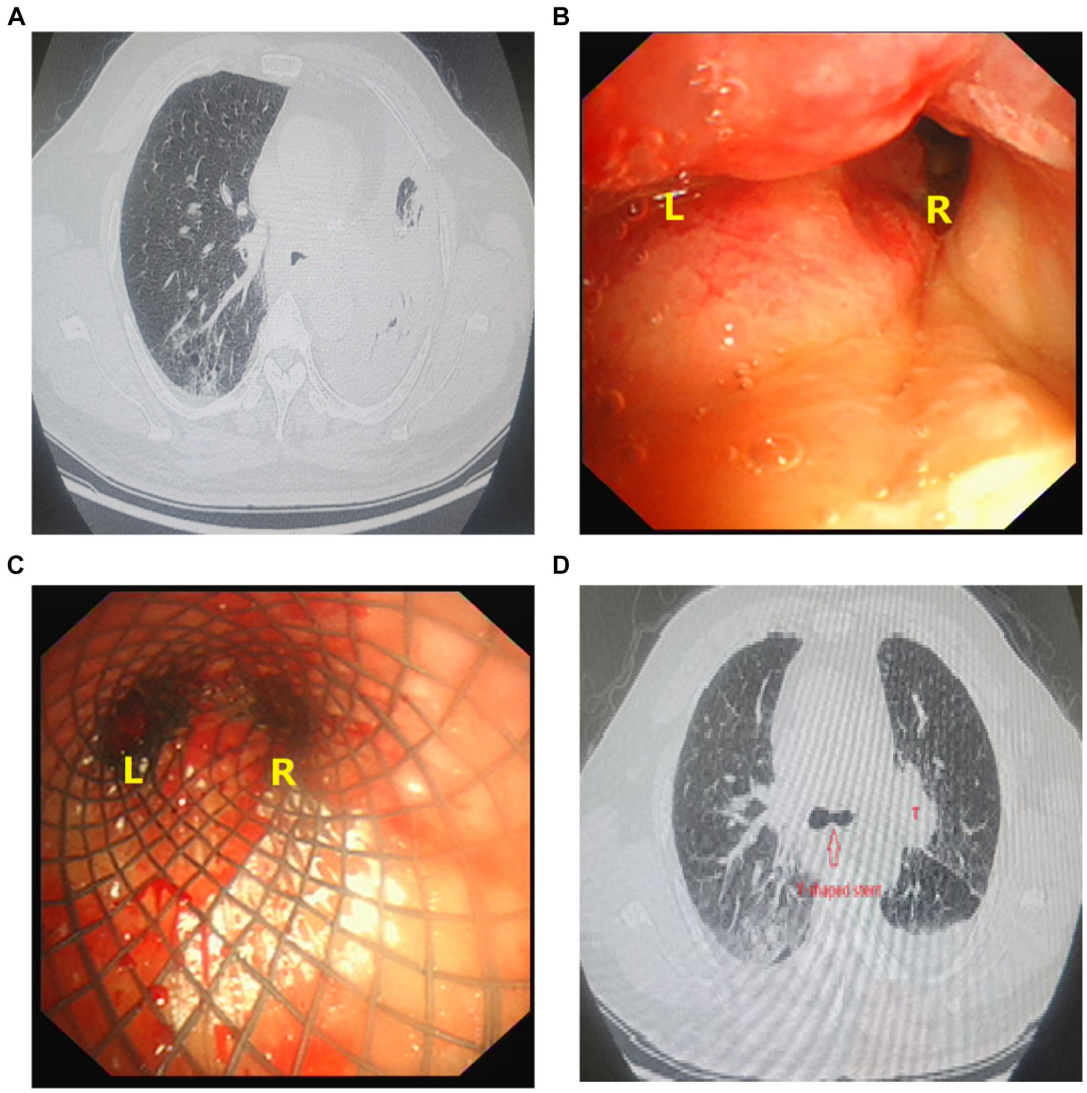
**(A)** Chest CT shows severe airway stenosis with left lung atelectasis. **(B)** Bronchoscopy shows complete obstruction of the left bronchus (L) due to tumor involving, right main bronchus (R) also involving by tumor with only about a quarter of the diameter remaining. **(C)** Y-shaped self-expanding metal covered stent was implanted within the patient’s airway. **(D)** Y-shaped self-expanding metal covered stent was implanted within the airway (), rustling in fully recover from left lung atelectasis. A tumor with a diameter of 5 cm in the left hilum (T).

A 71-year-old man (Identification number 357039) was admitted to our hospital presenting with extreme dyspnea for a day. Upon admission, chest CT scan and bronchoscopy found complete obstruction of the left bronchus due to tumor involving it, resulting in left lung atelectasis. The tumor simultaneously involved the trachea, carina, and right main bronchus with only about a quarter of the diameter remaining (Figures 2A,B). Following laser ablation and cryoablation, partial recanalization of the left and right main bronchi was achieved, and then a customized self-expanding Y-shaped metal covered stent (Micro-Tech Medical Company, Nanjing, China) was meticulously implanted in the patient’s airway, leading to full recover from left lung atelectasis (Figures 2C,D). After subsequent radiotherapy and other treatments, a substantial reduction in the size of the left hilar tumor was observed, and the stent was removed 2 months after implantation. The details of the stent implantation procedure are also described below.

Since the stent is individually designed according to the patient’s airway condition, the two patients can quickly adapt after stent implantation. The later complications mainly include the adhesion of secretions and the growth of granulation tissue on the stent, which require cleanup as needed.

### The details of procedure steps for precise implantation of Y-shaped stent

**Figure 3:**
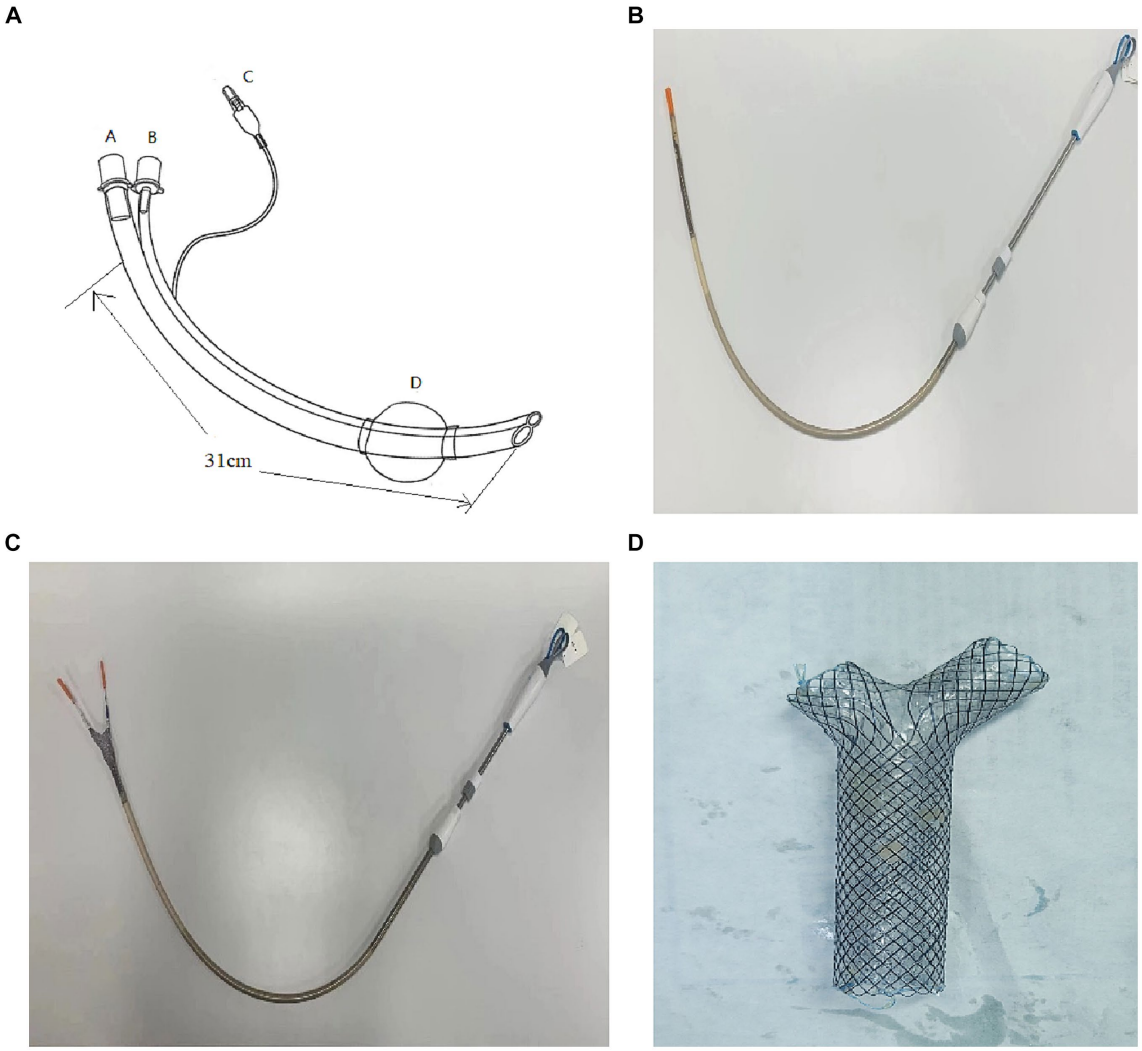
**(A)** Displays the innovative double lumen endotracheal intubation (outer diameter 16.0 mm, length 310.0 mm) designed for this procedure; tube A (operating tube, inner diameter 9.0 mm) serves as a pathway for the stent pusher (outside diameter: 8.0 mm); tube B (monitor tube, inner diameter 3.5 mm) provides a pathway for a flexible bronchoscopy (outside diameter less than or equal to 3.0 mm); D represents the balloon (inflated with air through channel C). **(B)** Illustrates the pusher (outside diameter: 8.0 mm) containing the Y-shaped stent. **(C)** Shows the bilateral branches of the Y-shaped self-expanding metal covered stent pushing out from the pusher. **(D)** Shows a Y-shaped self-expanding metal covered stent.

1. Insertion of the novel-designed double-lumen endotracheal intubation (Figure 3A, Chinese patent number 202321038543.3) to a predetermined position under the guidance of laryngoscope and bronchoscope after general anesthesia in patient.
2. The B tube is clamped and pure oxygen is supplied when connecting the A tube of this double-lumen endotracheal intubation to the ventilator in order to enhance the patient’s oxygen reserve.
3. Introducing a flexible bronchoscopy with an outer diameter less than 3.0 mm through tube B until a clear view of the carina is achieved.
4. Temporarily disconnecting the tube A from the ventilator, the pusher containing the Y-shaped stent (Figures 3B,C) was swiftly inserted into the tube A.
5. With guidance from the flexible bronchoscopy (within the tube B), the bilateral branches of the Y-shaped stent (Figure 3D) was released into the predetermined left and right bronchi from the pusher (within the tube A).
6. Removing the flexible bronchoscopy from the tube B, the Y-shaped stent was completely released from the pusher.

The stent implantation procedure takes only a few dozen seconds. If the whole process is calculated from anesthesia, intubation with a double-lumen endotracheal tube, stent implantation, to anesthesia recovery, the entire procedure takes less than 1 hour.

## Discussion

Esophageal-tracheal/bronchial fistula is a pathological connection between the esophagus and the trachea/bronchi, caused by various benign and malignant factors (11, 12). Malignant factors include advanced esophageal cancer, advanced lung cancer, mediastinal malignancy, and thyroid cancer. The incidence of this condition is about 5 ~ 15% in patients with esophageal cancer and approximately 1% in patients with lung cancer. Esophageal-tracheal/bronchial fistula allows gastric contents to enter the respiratory tract, leading to difficulty controlling pneumonia and a high mortality rate. Surgery is the preferred treatment, but some patients may be unable to undergo it due to poor physical condition. In such cases, implantation of airway covered stent, esophageal covered stent, or a combination of both can effectively seal the fistula, prevent gastric contents to enter the respiratory tract.

Severe airway stenosis is a life-threatening condition. Surgery is an effective treatment, but some patients cannot undergo it due to poor performance status or advanced tumors. In such cases, airway stent implantation is a valuable alternative (13, 14), which can quickly relieve breathing difficulties and allow for further treatments, and extend the survival time. After successful alleviation of stenosis, the stent can be safely removed, significantly improving the patient’s quality of life (15). A specific case described in the paper illustrates the effectiveness of this approach. In case 2, the patient’s stenosis was effectively relieved by following radiotherapy, enabling stent removal and leading to an improved quality of life.

Stent implantation is a viable treatment option for esophageal-tracheal/bronchial fistula and airway stenosis, particularly when adjacent to the carina. There are three main methods for tracheal Y-shaped stent implantation: flexible bronchoscopy combined with X-ray fluoroscopy guidance (3–5), rigid bronchoscopy combined with flexible bronchoscopy guidance (6–10), and laryngeal mask airway (LMA) combined with either flexible bronchoscopy or X-ray fluoroscopy guidance (16, 17). The first method involves using flexible bronchoscopy to insert separate guide wires into the left and right bronchi, followed by introducing a stent pusher through these guide wires. The pusher is guided to the desired position using X-ray, and the Y-shaped stent is released. However, this method has drawbacks such as radiation exposure and the risk of guide wire displacement and imprecise stent release. Improper placement of the Y-shaped stent can lead to serious consequences, such as asphyxiation. The second method utilizes rigid bronchoscopy, which requires specialized technical expertise. It operates with both the stent pusher and flexible bronchoscopy within the same channel of rigid bronchoscopy, potentially causing interference. Patients may also experience throat discomfort following rigid bronchoscopy. The third method combines LMA with either flexible bronchoscopy or X-ray guidance (16, 17). However, passing the stent pusher through the glottis can be challenging and may result in complications like irritation to the vocal cords, glottic edema, and bleeding. Additionally, if complications arise during stent implantation using only LMA, re-intubation becomes necessary, carrying significant risks.

In summary, each of the existing methods for tracheal Y-shaped stent implantation has its limitations and potential complications. To overcome the aforementioned shortcomings, we designed a double lumen endotracheal intubation for tracheal Y-shaped stent implantation under direct vision of flexible bronchoscopy. This approach offers several advantages: (1) the double lumen endotracheal intubation is thinner and more flexible compared to rigid bronchoscopy, making insertion easier and improving patient comfort; (2) separate channels for the flexible bronchoscope and stent pusher prevent interference; (3) the stent pusher enters through a thick channel, minimizing the risk of glottic damage, while the bronchoscope enters through a thin channel for precise monitoring. This innovative approach improves precision, safety, and patient comfort in tracheal Y-shaped stent implantation for esophageal-tracheal/bronchial fistula and severe airway stenosis. Additionally, it has the potential to update current operating standards and guidelines for this procedure.

## Data availability statement

The original contributions presented in the study are included in the article/supplementary material, further inquiries can be directed to the corresponding author.

## Ethics statement

The studies involving humans were approved by Ethics Committee of Beijing Millennium Temple Hospital affiliated to Capital Medical University. The studies were conducted in accordance with the local legislation and institutional requirements. Written informed consent for participation in this study was provided by the participants’ legal guardians/next of kin. Written informed consent was obtained from the individual(s), and minor(s)’ legal guardian/next of kin, for the publication of any potentially identifiable images or data included in this article.

## Author contributions

X-MD: Conceptualization, Data curation, Formal analysis, Methodology, Supervision, Validation, Writing – original draft, Writing – review & editing, Investigation, Project administration. Y-AD: Writing – original draft, Software. Y-FD: Data curation, Writing – review & editing. J-YC: Writing – review & editing. LL: Writing – review & editing. F-PR: Writing – original draft. JS: Writing – original draft.
